# Prospective clinical trial evaluating vulnerability and chemotherapy risk using geriatric assessment tools in older patients with lung cancer

**DOI:** 10.1111/ggi.13781

**Published:** 2019-11-20

**Authors:** Yukari Tsubata, Yohei Shiratsuki, Takae Okuno, Akari Tanino, Mika Nakao, Yoshihiro Amano, Takamasa Hotta, Megumi Hamaguchi, Tamio Okimoto, Shunichi Hamaguchi, Noriaki Kurimoto, Yumi Nishiyama, Tomohiro Kimura, Haruko Iwata, Shusaku Tsumoto, Takeshi Isobe

**Affiliations:** ^1^ Division of Medical Oncology and Respiratory Medicine, Department of Internal Medicine, Faculty of Medicine Shimane University Izumo Japan; ^2^ Division of Medical Service Shimane University Hospital Izumo Japan; ^3^ Toshiba Medical Systems Corporation Tochigi Japan; ^4^ Department of Medical Informatics, Faculty of Medicine Shimane University Izumo Japan

**Keywords:** chemotherapy, *EGFR* mutation, geriatric assessment, geriatric oncology, lung cancer

## Abstract

**Aim:**

In Japan, the number of older patients with cancer has been increasing. Assessment of performance status, cognitive function and social background is necessary for the treatment of older patients. The aims of the present study were: (i) to establish an evaluation system using electronic medical records; and (ii) to distinguish older patients as fit versus vulnerable or frail according to a geriatric assessment (GA) system score.

**Methods:**

We incorporated GA tools in our electronic medical records system and carried out comprehensive assessments for patients with newly diagnosed lung cancer aged ≥65 years. The decision about primary treatment followed consultation with the clinical team and was not guided by GA scores. Subsequent treatment and outcomes were recorded.

**Results:**

A total of 100 patients had completed GA. The average age was 75 years (range 65–94 years). Regarding GA results, 63% were positive on the Comprehensive Geriatric Assessment 7, 39% on the Vulnerable Elderly Survey‐13 and 84% on the Geriatric 8. The percentage of vulnerable patients (positive on all three GA) was significantly higher in the non‐standard therapy group (*n* = 19) than in the standard therapy group (*n* = 81; 78.9% *vs* 21.0%, *P* < 0.001). Among vulnerable patients who received standard therapy, 47% discontinued chemotherapy as a result of toxicity. Even if a patient was considered vulnerable based on GA scores, chemotherapy is possibly safe for those with *EGFR* mutations.

**Conclusions:**

We confirmed the feasibility of this system. During decision‐making for older patients with cancer, a combination of GA helps prevent undertreatment or overtreatment. **Geriatr Gerontol Int 2019; 19: 1108–1111**.

## Introduction

Japan is a super‐aged society that is ranked as one of the developed countries in terms of average life expectancy, proportion of older people and speed of aging. According to a report from the Statistics Bureau of the Ministry of Internal Affairs and Communications in Japan, individuals aged ≥65 years constituted 28.1% of the total population in 2018, and this figure is predicted to exceed 30% by 2025.^1^ In contrast, the most common cause of mortality among Japanese individuals for >30 years has been malignant neoplasm. The mortality for malignant neoplasm continues to rise. Details on decision‐making for older patients with cancer are described in the National Comprehensive Cancer Network Clinical Practice Guidelines in Oncology (NCCN Guidelines) for Older Adult Oncology Version 1.2019.^2^ The guidelines use a flow chart to explain that a prediction of prognosis for a patient is made first. Next, a determination about cognitive function, in terms of whether or not the patient understands his or her disease state, and determination and acceptance of the treatment strategy are made. Afterwards, the patient's goals for treatment are discussed and treatment preferences are confirmed. A risk assessment is subsequently carried out in the event of chemotherapy. Geriatric assessment (GA) involves domains specific to older adults, such as cognitive function and activities of daily living, that are known to be associated with adverse events and survival. Evidence supporting the use of GA for the evaluation and management of vulnerabilities in older cancer patients has been increasing.^3^, ^4^, ^5^ The American Society of Clinical Oncology guidelines for geriatric oncology provide guidance regarding practical assessment and management of vulnerabilities in older patients receiving chemotherapy.^6^

However, in Japan, there are extremely few geriatric specialists in oncology compared with Western countries. Validation of many screening tools among Japanese individuals has not been carried out, and they are not in widespread use. Therefore, many cases of undertreatment, in which the intensity of a treatment is inappropriately lowered simply due to advanced chronological age, or overtreatment, in which treatment provided to young people is carried out without taking into consideration the risks of chemotherapy in practical settings, might be occurring.

At Shimane University Hospital in Shimane, Japan, we have developed ways to carry out screening by first creating GA screening tools in electronic medical records (EMR) in cooperation with the Department of Medical Informatics. Using this system, we carried out a prospective clinical trial to evaluate vulnerability and chemotherapy risks in older patients with newly diagnosed lung cancer at our hospital.

## Methods

### *Patients*

The main eligibility criteria were age ≥65 years and histologically or cytologically proven lung cancer (small cell or non‐small cell) treated at Shimane University Hospital. Patients diagnosed with geriatric syndrome, especially apparent dementia, were excluded. We planned to use a sample size of 100 patients, because approximately 50 patients are newly diagnosed with lung cancer at our hospital each year. This study was approved by the ethics committee of Shimane University (No. 1718). As all data were from general medical treatment, the committee determined that written consent from each participant was not required.

### *GA assessment and data collection*

We incorporated several GA tools (Comprehensive Geriatric Assessment 7 [CGA7; Appendix S1, in Japanese], Vulnerable Elderly Survey‐13 [VES‐13; Appendix S2, in Japanese], Geriatric 8 [G8; Appendix S3, in Japanese] and the Charlson Comorbidity Index [CCI; Appendix S4, in Japanese]) in our EMR through corroboration with the Department of Medical Informatics, Shimane University. One nurse carried out the multifaceted and comprehensive assessments.

CGA7 is a simplified geriatric assessment tool comprised of seven items.^7^ We defined cases that were not perfect as positive. VES‐13 is a well‐known GA tool recommended by the National Comprehensive Cancer Network guidelines.^8^ We used the Japanese version and defined ≥3 points as positive. G8 consists of eight items, including age and body mass index. It reflects not only nutritional status, but also prognosis.^9^, ^10^, ^11^ We defined ≤14 points as positive. Finally, we defined patients who were positive for three GA as vulnerable.

We also used the CCI for older patients scheduled to receive chemotherapy.^12^ The objective of this tool is to clarify comorbidities and evaluate the risks associated with chemotherapy. The domains explored and the scales used are described in the Appendix (Appendix S1–S4, in Japanese).

### *Treatment procedure and data collection*

Decisions regarding primary treatment were based on consultation with the clinical team; they were not guided by GA results. We recorded patient background (age, sex, performance status, histological type and clinical stage), subsequent treatment regimen and adverse events. We defined the primary treatment recommended by the 2017 Guidelines for Diagnosis and Treatment of Lung Cancer by the Japanese Lung Cancer Society as standard treatment.^13^ All other treatment regimens were classified as non‐standard treatment. Adverse events were reported throughout the first‐line treatment period and assigned grades based on the National Cancer Institute Common Terminology Criteria for Adverse Events version 4.0. We also recorded whether the first‐line treatment was discontinued as a result of any toxicity.

### *Statistical analysis*

All statistical analyses were carried out using sas software (SAS Institute, Cary, NC, USA). *P* < 0.01 was considered statistically significant in all analysis.

## Results

### *Patient characteristics*

From April 2015 to December 2016, a total of 100 patients aged ≥65 years were enrolled. Patient characteristics are summarized in Table 1. The median age was 75 years (range 65–94 years); 39% of patients were aged ≥80 years. Most patients (73%) were men. The Eastern Cooperative Oncology Group performance status score was 0 in 33% of patients, 1 in 47%, 2 in 14% and 3 in 6%. Histological types included non‐small cell lung cancer in 86% of patients (adenocarcinoma, 57%; squamous cell carcinoma, 20%; and other [not otherwise specified], 9%) and small cell carcinoma in 14% of patients. The clinical stage was IIIB/IV in 49% of patients; the remaining 51% had stage I–IIIA disease.

**Table 1:** Patient characteristics

		No. patients (total *n* = 100)	Radiotherapy and/or chemotherapy, BSC (*n* = 83)
Age (years)	Median (range)	75 (65–94)	73 (65–88)
Sex	Male/female	73/27	62/21
ECOG‐PS	0/1/2/3	33/47/14/6	24/42/11/6
Histology	Adenocarcinoma	57	48
	Squamous cell carcinoma	20	17
	Others†	9	4
	Small cell	14	14
Stage (UICC ver. 7.0)	IA–IIIA (curative)	50	17
	IA–IIIA (not curative)		16
	IIIB/IV	50	50

† Adenosquamous, large cell carcinoma and not otherwise specified.

BSC, best supportive care; ECOG‐PS, Eastern Cooperative Oncology Group ‐ performance status; UICC, Union for International Cancer Control.

The proportion of patients considered for concurrent chemoradiotherapy, chemotherapy including molecular targeted therapy and best supportive care (BSC) as first‐line treatment was 83%; the remaining 17% were considered for complete resection.

### *Association between treatment decision and GA scores*

**Figure 1:**
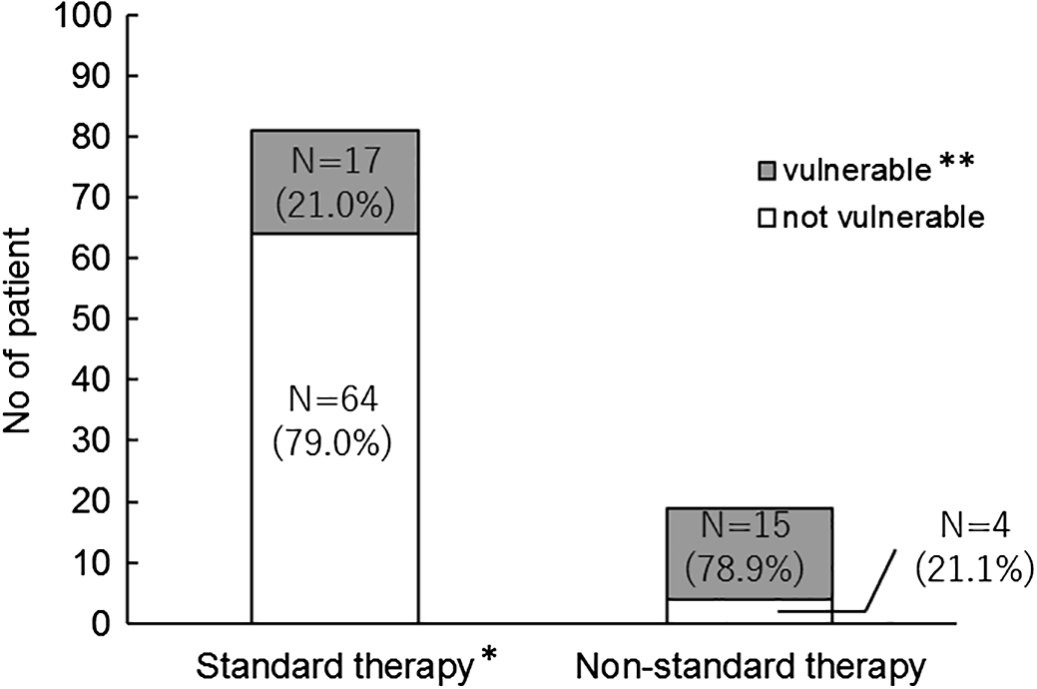
Geriatric assessment results for patients receiving standard and non‐standard therapy. A higher proportion of the non‐standard therapy group was considered vulnerable compared with the standard therapy group (78.9% *vs* 21.0%, *P* < 0.001). *Standard treatment was defined as the primary treatment recommended in the 2017 Guidelines for the Diagnosis and Treatment of Lung Cancer by the Japanese Lung Cancer Society. **Patients with all geriatric assessments being positive were considered vulnerable.

All patients were evaluated using GA tools. On the CGA7, 63% of patients were categorized as positive, whereas 39% were positive on the VES‐13 and 84% on the G8. The proportion of vulnerable patients with all three GA being positive was significantly higher in the non‐standard therapy group (*n* = 19) than in the standard therapy group (*n* = 81; 78.9% *vs* 21.0%, *P* < 0.001; Fig. 1). Among patients being considered for concurrent chemoradiotherapy, chemotherapy including molecular targeted therapy and BSC, the proportion of patients categorized as positive was 47% on the CGA7 (39 patients), 24.1% on the VES‐13 (20 patients) and 69.9% on the G8 (58 patients). There were 28 patients (33.7%) with three positive GA, a subgroup considered to be vulnerable.

### *Association between treatment progress and GA scores*

**Figure 2:**
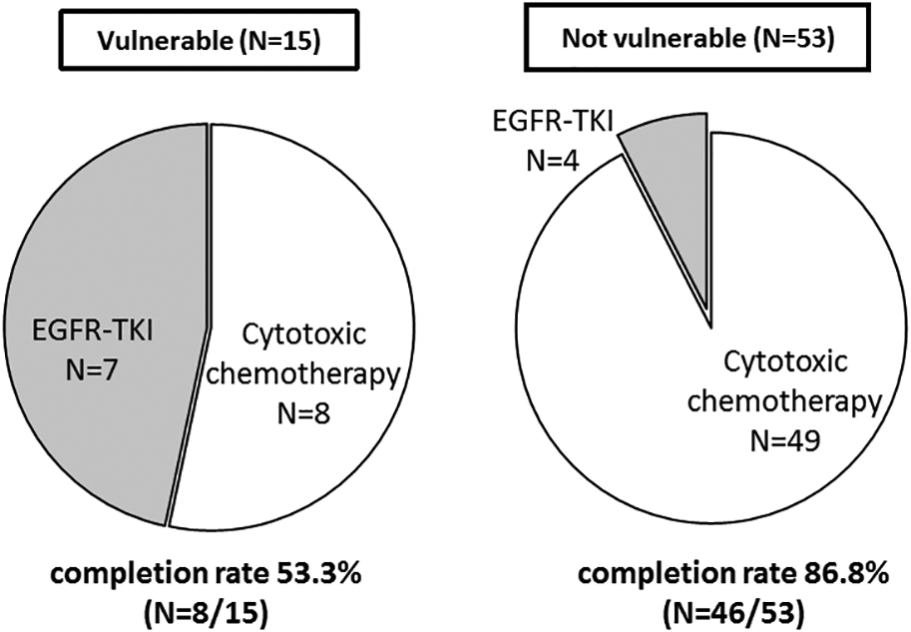
Association between chemotherapy regimen and geriatric assessment scores. BSC, best supportive care; ECOG‐PS, Eastern Cooperative Oncology Group performance status; EGFR‐TKI, epidermal growth factor receptor–tyrosine kinase inhibitor.

We assessed the associations between treatment progress and patient background (including GA scores) among 68 patients who underwent chemotherapy (Table 2). A total of 53 patients were classified as vulnerable, and 15 patients were classified as fit. We defined completion of treatment as continued use of epidermal growth factor receptor (EGFR) – tyrosine kinase inhibitors for 3 months or four cycles of cytotoxic chemotherapy. The chemotherapy completion rate was 86.8% (46/53 patients) in the not vulnerable group (Fig. 2). In contrast, the chemotherapy completion rate decreased to 53.3% (8/15 patients) in the vulnerable group. Among 14 patients in the fit and vulnerable groups that were assessed as high risk based on the CCI, 13 (92.9%) withdrew from chemotherapy as a result of toxic events. Regarding patients on BSC, all were negative for *EGFR* mutations and were considered vulnerable based on GA scores.

**Table 2:** Patient characteristics (chemotherapy)

		Vulnerable‡ (*n* = 15)	Not vulnerable (*n* = 53)
Age (years)	Median (range)	83 (66–87)	73 (65–88)
Sex	Male/female	10/5	47 / 11
ECOG‐PS	0/1/2/3	2/7/4/2	20/33/4/1
Histology	Adenocarcinoma (*EGFR* mutation +/−)	9 (3/6)	29 (8/21)
	Squamous cell carcinoma	1	11
	Others†	0	4
	Small cell	5	9

† Adenosquamous, large cell carcinoma and not otherwise specified.

‡ All three geriatric assessments were positive was confirmed vulnerable.

BSC, best supportive care; ECOG‐PS, Eastern Cooperative Oncology Group ‐ performance status.

## Discussion

Although the number of older patients with cancer has been increasing in Japan, it is not common to use GA for decision‐making about treatment; guidelines do not exist. In the present study, we confirmed the feasibility of carrying out GA screening among older patients with lung cancer by introducing GA into our EMR system. In addition, we identified an association between GA scores and the onset of adverse events with treatment.

GA consist of a compilation of validated tools for assessing specific domains known to be associated with survival or adverse outcomes in older patients with cancer. Several studies have shown that GA can identify older patients with cancer at increased risk for mortality.^14^, ^15^ The systematic review by Hamaker *et al*. reported that in the 35 studies that met the inclusion criteria, the oncological treatment plan was altered primarily to a less intensive treatment option after a geriatric evaluation.^16^ However, because GA have not become widespread throughout Japan, it is very difficult to carry out GA in an outpatient setting. We used three GA tools (CGA7, VES‐13 and G8) in the present study to cover all items related to seven factors (function, comorbidity, cognition, depression, nutrition, polypharmacy and social support) that are indispensable to the evaluation of older patients with cancer that can be used in a short period of time. We prospectively registered patients with lung cancer aged ≥65 years. Only one patient refused a usability test that was a component of GA (data were not shown). For both the medical staff and patients, GA tools were considered user friendly. Generally, we believe the advantages of incorporating these assessment tools in the EMR system are that any available member of the medical staff can carry out the evaluation, because responses need to be confirmed automatically at the time of EMR registration, resulting in screening that can occur quickly.

In the present study, the decision on primary treatment followed consultation with the clinical team and was not guided by GA. Among the seven patients who chose BSC as a result of a lung cancer conference, all were vulnerable (all three GA scores were positive). Judgment and GA results were based on the daily life medical treatment of the chief physician, so it is possible that a decision‐making tool does not make a difference. Thus, treatment decisions in the clinical setting by medical doctors and GA results might be similar, especially for patients on BSC. However, for patients undergoing chemotherapy, the rate of withdrawal from treatment as a result of adverse events was very high (46.7% in vulnerable group), and the use of GA was useful for predicting toxicity. The American Society of Clinical Oncology guidelines suggest using either the Cancer and Aging Research Group^17^ or Chemotherapy Risk Aging Score for High‐Age patients^18^ tool to obtain specific estimates on the risk of chemotherapy toxicity.^6^ Although we tried to predict adverse outcomes for patients receiving chemotherapy using the Chemotherapy Risk Aging Score for High‐Age patients score in the present study, the Chemotherapy Risk Aging Score for High‐Age patients score does not include items relevant to molecular targeted therapy and immune checkpoint inhibitors, which are of critical importance for treating lung cancer. Thus, the development of an appropriate risk assessment tool for current lung cancer treatment is required.

GA tools have not become widespread throughout Japan, but more effective and safer treatment of older patients with cancer is possible by stratifying older patients with cancer and carrying out timely functional assessments as well as appropriate GA when devising a treatment regimen tailored to each patient's individual condition and providing a support system. We are planning a multicenter, prospective study with hospitals throughout Japan using the same system. Our goal is to clarify whether it is possible to distinguish older patients requiring multidisciplinary support and intervention during cancer treatment using GA results. Furthermore, verification of the associations between GA results, treatment response rate and overall survival is necessary. These activities will contribute to providing precise treatment and care for older patients with cancer.

## Disclosure statement

The authors declare no conflict of interest.

## Supporting information


**Appendix S1**. Comprehensive Geriatric Assessment (CGA) 7.

**Appendix S2**. Vulnerable Elderly Survey (VES)‐13.

**Appendix S3**. Geriatric (G) 8.

**Appendix S4**. Charlson Comorbidity Index (CCI).
