# Supplementary material for: Prospective clinical trial evaluating vulnerability and chemotherapy risk using geriatric assessment tools in older patients with lung cancer
Source: Geriatr Gerontol Int. 2019 Nov 20;19(11):1108–11. doi: 10.1111/ggi.13781 (PMC6899794; doi:10.1111/ggi.13781)
Supplement: Supplementary file 1 — Appendix S1. Comprehensive Geriatric Assessment (CGA) 7. [file GGI-19-1108-s001.docx]

Doc S1

CGA7

１．外来または問診時に、患者の挨拶を待つ

□自分から進んで挨拶する（1点）　　　□返事のみ、または反応なし

２．「これからいう言葉を繰り返してください。（桜、猫、電車）」

（「あとでまた聞きますから覚えておいてくださいね」）

□可能（1点）　　　□不可能

３．「ここへどうやって来ましたか？」

□自分でバス、電車、タクシー、自家用車を使って移動（1点）□付き添いが必要

４．「先ほど（2）覚えていただいた言葉を言ってください。」

□ヒントなしで全部正解（1点）　　　□それ以外

５．「お風呂は一人で入って、洗うのも手助けはいりませんか？」

□入浴自立（1点）　　　□部分介助または全介助

６．「トイレに間に合わず、漏らしたりすることはありませんか？」

「トイレに行けないときは尿瓶を自分で使えますか？」

□失禁なし、尿瓶使用は自立（1点）　　　□それ以外

７．「自分は無力だと思うことはありますか？」

□いいえ（1点）　　　□はい

合計スコア：　　　　点
