# Supplementary material for: Prospective clinical trial evaluating vulnerability and chemotherapy risk using geriatric assessment tools in older patients with lung cancer
Source: Geriatr Gerontol Int. 2019 Nov 20;19(11):1108–11. doi: 10.1111/ggi.13781 (PMC6899794; doi:10.1111/ggi.13781)
Supplement: Supplementary file 2 — Appendix S2. Vulnerable Elderly Survey (VES)‐13. [file GGI-19-1108-s002.docx]

Doc S2

　VES-13

１．年齢　　　　　歳　　スコア：　　　　点（75～84歳：1点、　85歳以上：3点）

２．一般に、同年齢の他人と比較して、あなたの健康状態はどのようだと言えますか？

□不良　（１点）

□普通　（１点）

□良い　　（0点）

□非常に良い（0点）

□極めて良い（0点）　　　　　　　　　　　　スコア：　　　　点

３．下記の身体的活動に関して、平均してどの程度の困難を感じていますか？

　　　　　　　　　　　　　　 なし　 少し　 幾分　 たくさん　できない

　　　　　　　　　　　　　　　　　　　　　　　　　　　　　　　　　　　　　　　　　（1点）　（1点）

1. かがむ、しゃがむ、または

ひざまずく　　　　　　　　 □ □ □ □ □

1. 重さ4~5㎏ほどの物を

持ち上げるまたは運ぶ　　 　 □ □ □ □ □

1. 肩より上で両腕を伸ばす

または広げる　　　　　　 　□ □ □ □ □

1. 文字を書くまたは小さな

ものを握るまたはつかむ　 　□ □ □ □ □

1. 400ｍを歩く □ □ □ □ □
2. 重労働の家事、（床をゴシゴシ

洗う、または窓を洗うなど）　□ □ □ □ □

　　　　　　　　　　　　　　　　　　　　　　　　　　スコア：　　　　点

スコア：質問3a~fにおける※の回答についてそれぞれ1点。いくつチェックがついても

最大2点。

４．あなたの健康状態または身体的状況が原因で、困難が生じることはありますか?

**a.　個人的な用品（トイレ用品または医薬品）の買い物**

□　はい　→　買い物を手伝ってもらいますか？　　 　　□はい　　□いいえ

□　いいえ（困難なくできる）

□　しない　→　健康状態が原因ですか？　　　　　　　　□はい　　□いいえ

**b.　金銭管理（出費または支払いの証跡をつけるなど）**

□　はい　→　金銭管理を手伝ってもらいますか？ 　　　 □はい　　□いいえ

□　いいえ（困難なくできる）

□　しない　→　健康状態が原因ですか？　　　　　　 □はい　 □いいえ

**c.　部屋の中を歩く**（杖または歩行器の使用は可とする）

□　はい　→　歩行を手伝ってもらいますか？　 □はい □いいえ

□　いいえ（困難なくできる）

□　しない　→　健康状態が原因ですか？　 □はい 　□いいえ

**d.　軽い家事労働（皿洗い、整理整頓または簡単な掃除など）**

□　はい　→　家事労働を手伝ってもらっていますか？ 　 □はい 　□いいえ

□　いいえ（困難なくできる）

□　しない　→　健康状態が原因ですか？ 　□はい 　□いいえ

**e.　入浴またはシャワー**

□　はい　→　入浴またはシャワーを手伝ってもらっていますか？

　　　　　　　　　　　　　　　 □はい 　□いいえ

□　いいえ（困難なくできる）

□　しない　→　健康状態が原因ですか？　 □はい 　□いいえ

↑

この列の「はい」に1個でもチェックがあれば4点

　　　　　　　　　　　　　　　　　　　　　　　　　　スコア：　　　　点

スコア（4か所）の合計：　　　　点

（3点以上で脆弱性あり）
