# Supplementary material for: Prospective clinical trial evaluating vulnerability and chemotherapy risk using geriatric assessment tools in older patients with lung cancer
Source: Geriatr Gerontol Int. 2019 Nov 20;19(11):1108–11. doi: 10.1111/ggi.13781 (PMC6899794; doi:10.1111/ggi.13781)
Supplement: Supplementary file 3 — Appendix S3. Geriatric (G) 8. [file GGI-19-1108-s003.docx]

Doc S3

　G8

スコアの合計：　　　　点

（14点以下で脆弱性あり）
