# Supplementary material for: Prospective clinical trial evaluating vulnerability and chemotherapy risk using geriatric assessment tools in older patients with lung cancer
Source: Geriatr Gerontol Int. 2019 Nov 20;19(11):1108–11. doi: 10.1111/ggi.13781 (PMC6899794; doi:10.1111/ggi.13781)
Supplement: Supplementary file 4 — Appendix S4. Charlson Comorbidity Index (CCI). [file GGI-19-1108-s004.docx]

Doc S4

Charlson Co-morbidity Index (CCI)

１．心筋梗塞の既往（ECGの変化のみは含まない）

□はい（1点）　　　□いいえ

２．うっ血性心不全

□はい（1点）　　　□いいえ

３．末梢血管疾患（大動脈が6cm以上も含む）

□はい（1点）　　　□いいえ

４．認知症

□はい（1点）　　　□いいえ

５．慢性肺疾患

□はい（1点）　　　□いいえ

６．膠原病

□はい（1点）　　　□いいえ

７．消化性潰瘍

□はい（1点）　　　□いいえ

８．糖尿病

　　　□はい、合併症あり（2点）　　□はい、合併症なし（1）　　□いいえ

９．中等度から重症の腎疾患

□はい（2点）　　□いいえ

１０．脳血管疾患

□片麻痺（2点）　　□軽度または後遺症の無いTIA（1）　　　□いいえ

１１．白血病（急性か慢性）

□はい（2点）　　　□いいえ

１２．悪性リンパ腫

□はい（2点）　　　□いいえ

１３．悪性腫瘍（診断後5年以上の疾患は除外）

□遠隔転移あり（6点）　　□遠隔転移なし（2点）　　　□いいえ

１４．肝疾患

　　　□中等度から重症（3点）　　□軽症（慢性肝炎を含む）（1点）　　□いいえ

１５ ．AIDSである（HIVの有無は問わない）

□はい（6点）　　　□いいえ

合計スコア：　　　　点
